# The attitude-behavior gap and the role of infrastructure availability in waste sorting at mass sport events in Vietnam

**DOI:** 10.3389/fspor.2026.1782725

**Published:** 2026-06-18

**Authors:** Phuc Van Nguyen, Ngoc Quang Dinh, Thu Khanh Dinh

**Affiliations:** 1 Bac Ninh Sport University of Vietnam, Bac Ninh, Vietnam; 2 Institute of Sport Science, Technology, and Foreign Affairs, Bac Ninh Sport University of Vietnam, Bac Ninh, Vietnam

**Keywords:** attitude-behavior gap, infrastructure availability, mass sport events, PLS-SEM, source-separated waste sorting

## Abstract

This study explores the mechanisms underlying the formation of source-separated municipal solid waste (MSW) sorting behavior among participants at mass sport events (MSEs) in Vietnam. By integrating the Theory of Planned Behavior (TPB) and the Attitude-Behavior-Context (ABC) framework, the research analyzes the correlations between environmental perception, attitudes, and the role of Infrastructure Availability. Data were collected from 460 participants across MSEs in Hai Phong, Bac Giang, and Thanh Hoa. The situational findings reveal a significant paradox: despite high levels of recorded environmental perception and positive attitudes, the actual waste sorting rate reached only 15.87%. Results from Partial Least Squares Structural Equation Modeling (PLS-SEM) indicate that environmental perception is the most significant antecedent in forming positive attitudes (*β* = 0.399, *p* < 0.001). The relationship between attitude and behavior is statistically significant but recorded the lowest effect size (*β* = 0.129, *p* < 0.05), confirming the existence of a persistent “attitude-behavior gap” in the event context. Notably, Infrastructure Availability emerged as the strongest direct predictor of behavior (*β* = 0.180, *p* < 0.001), surpassing the influence of individual attitudes. The study rejects the moderating role of infrastructure (*p* = 0.883), asserting that deficiencies in physical facilities (e.g., lack of sorting bins and visual guidance) function as independent “hard barriers” rather than mere catalytic factors. These findings suggest a critical shift in management: beyond awareness-raising communication, event organizers must prioritize optimizing the physical context and reducing “effort costs” through the synchronization of on-site technical infrastructure.

## 1 Introduction

Sustainable municipal solid waste (MSW) management remains a core challenge intertwined with the Sustainable Development Goals (SDGs) amidst rapid globalization and urbanization (1). In recent years, mass sport events (MSEs) have become a focal point of environmental concern due to their high participant density within short timeframes. This often leads to a surge in waste generation, exerting severe pressure on local environmental management systems (2–5). Globally, waste generation at major events is estimated to range from 0.25 to over 7 kg per spectator per day, creating immediate stress on local collection, sorting, and disposal capacities (6, 7). In Vietnam, recyclable materials can account for up to 30% of waste at these events, yet the potential for a circular economy remains unexploited due to low source-separation rates (8). Notably, although participants’ environmental perception is often high, their actual behavior remains disproportionately low, reflecting a pronounced “attitude–behavior gap” (9).

### 1.1 Theoretical foundations and the attitude—behavior gap

To explain this phenomenon (10), Theory of Planned Behavior (TPB) is frequently employed, emphasizing attitude as a critical determinant of intention and behavior. Complementarily, the Value–Belief–Norm (VBN) theory (11) asserts that environmental perception serves as the foundation for forming positive attitudes. However, the persistent attitude–behavior gap in practice suggests that contextual factors can diminish the predictive power of attitudes (1). A recent meta-analysis of 46 studies (50 independent samples, *N* = 30,250) confirmed that waste separation behavior is most strongly associated with perceived behavioral control, intention, infrastructure, publicity and education, and attitude (12). Significantly, the same meta-analysis identified infrastructure as one of the strongest contextual enablers, underscoring that deficiencies in physical facilities can substantially undermine the translation of positive attitudes into action.

Recent evidence highlights strong “behavioral leakage” during events, where positive perceptions and attitudes fail to translate into corresponding actions (7). Studies in developing economies, such as China (13) and Saudi Arabia (14), consistently show high environmental intentions alongside low execution rates. Extended TPB research further indicates that perceived deficiencies in policy support or inadequate infrastructure reduce Perceived Behavioral Control (PBC), weakening the intention–behavior link (15). Within the broader context of sustainable consumption (16), using a TPB–ABC integration model on survey data from 534 consumers in China, demonstrated that while internal motivations (attitudes, subjective norms, and perceived behavioral control) significantly influence sustainable consumption behaviors, negative contextual factors—including lack of infrastructure—primarily affect recycling and resource conservation behavior, often indirectly shaping individual attitudes. This finding suggests that in developing countries, the absence of supportive infrastructure may not only hinder behavioral execution directly but may also weaken the formation of pro-environmental attitudes over time.

### 1.2 Recent empirical evidence in sustainable sport management

Within the specific domain of sustainable sport management, research has expanded considerably in recent years. A comprehensive bibliometric review of 393 journal articles covering the years 2000–2024 identified three primary thematic clusters: environmental sustainability, economic impacts, and social impacts, with a notable recent surge in research on technological innovations (17). However, the review critically notes that the literature is dominated by North American contexts, indicating a significant geographical gap. This finding reinforces the urgent need for empirical studies from developing countries, particularly in Southeast Asia, where mass sport events are increasingly popular but waste management infrastructure remains underdeveloped.

In sport spectator contexts, TPB has been extensively applied to understand recycling intentions. McCullough (18) utilized TPB to examine sport spectator recycling behaviors during large-scale sporting events, finding that recycling at such events has unique nuances that differentiate it from household or workplace settings. Similarly, (19) demonstrated that subjective norms and attitudes significantly predict recycling intentions among sport spectators, while perceived behavioral control becomes particularly salient in event contexts where time pressure and crowd dynamics prevail.

More recently, (20), in a comparative study involving more than 1,400 respondents, examined the relationship between pro-environmental attitude and pro-environmental behavior among emotionally involved sport fans and socially conscious university students. Their results showed that while sport fans exhibited higher average levels of both pro-environmental attitude and behavior, the influence of attitude on behavior was actually stronger for university students—the group with a more cognitive and intrinsic link to environmental sustainability. This finding suggests that the nature of an individual's connection to environmental issues (emotional vs. cognitive) moderates the strength of the attitude—behavior relationship, a nuance that has important implications for event-based environmental interventions.

Braksiek et al. (21) empirically tested TPB among sports club members and found that attitudes, subjective norms, and perceived behavioral control significantly predict intentions for environmentally friendly behavior, yet actual behavior lags behind—a gap that persists across genders and sports. Their study underscores that even in organized sport settings, internal psychological factors alone are insufficient to ensure pro-environmental action. Voráček et al. (22) explored the gap between attitudes and purchasing behavior regarding sustainable outdoor sports apparel, documenting a marked discrepancy: while consumers express strong pro-environmental attitudes, actual purchase rates of sustainable products remain low, highlighting the role of contextual barriers such as product availability, price sensitivity, and habitual consumption patterns. These findings collectively suggest that in sport-related contexts, the attitude–behavior gap is robust and that external, situational factors warrant greater analytical attention.

### 1.3 The role of infrastructure and the need for theoretical integration

Despite the growing body of research, a critical theoretical gap persists: the role of infrastructure availability in shaping waste sorting behavior at mass sport events remains under-theorized and under-empirically tested, particularly in developing countries. Most existing TPB-based studies in sport management (18, 21) have not explicitly modeled infrastructure as a direct predictor or moderator of actual sorting behavior at mass events. Similarly, studies on sustainable consumption in sport, e.g., Voráček et al. (22) highlight contextual barriers but rarely quantify them alongside psychological antecedents within a unified SEM framework.

The Attitude–Behavior–Context (ABC) model (23) provides a valuable complementary lens. According to the ABC model, behavior is a function of the interaction between individual attitudes and contextual conditions. In supportive contexts, attitudes strongly predict behavior; in unsupportive contexts, even highly positive attitudes fail to translate into action. However, the ABC model has been predominantly applied in household recycling settings in developed countries. Recent applications in developing contexts suggest that contextual factors may operate differently. For instance, (24), extending TPB with moral-psychological and contextual factors in South Korea, found that the Convenience of Available Recycling Infrastructure (CARI) had significant positive effects on recycling intentions, underscoring the importance of accessible infrastructure in sustainable waste management behavior. Notably, their study treated infrastructure convenience as a direct antecedent of intention rather than a moderator of the attitude–behavior relationship, reflecting a conceptual ambiguity that the present study seeks to address.

In Vietnam, the “high awareness—low behavior” paradox is distinctly observed within MSEs. Despite most participants possessing high education levels and positive environmental attitudes (25), actual waste sorting behavior consistently falls short of expectations (26). A recent study on university sport events in the Global South similarly concluded that event attendees exhibit a lack of pro-environmental behavior, resulting in negative environmental impacts, with minimal implementation of proper environmental management particularly regarding waste disposal and awareness raising (27). This situation raises a core research question: Which factors hinder the transformation of positive attitudes into pro-environmental behaviors in the context of MSEs?

This gap can be explained through the role of Infrastructure Availability. In sporting events—where behaviors are rapid, spontaneous, and subject to crowd pressure—a shortage of sorting bins, inconvenient placement, or inconsistent guidance increases “friction cost”. Conversely, optimized infrastructure facilitates smoother behavioral execution (28). Drawing on the ABC model, infrastructure may have two roles: a direct effect on behavior, and a moderating effect on the attitude–behavior relationship.

### 1.4 Research gaps, objectives, and hypotheses

Despite the valuable contributions of existing research, several critical gaps remain. First, most studies on pro-environmental behavior in sport have focused on intentions rather than actual behavior, largely due to the methodological challenges of measuring on-site actions. Second, the dual role of infrastructure—as both a direct predictor and a moderator of the attitude–behavior relationship—has not been empirically tested in the context of mass sport events in developing countries. Third, the applicability of the ABC model's moderation hypothesis in low-infrastructure settings remains untested. Fourth, the geographical bias in sustainable sport management research toward North American and European contexts means that findings may not generalize to countries with different cultural norms, economic conditions, and waste management systems.

Therefore, this study aims to: Integrate TPB and the ABC model to explain the attitude–behavior gap in MSEs; test both the direct and moderating effects of Infrastructure Availability on waste sorting behavior; provide empirical evidence from Vietnam, a developing country with rudimentary on-site waste management systems.

Based on the integrated framework, we propose:

H1: Environmental perception has a positive effect on participants' environmental attitudes.

H2: Environmental attitude has a positive effect on participants' waste sorting behavior.

H3a: Infrastructure availability has a direct positive impact on participants' waste sorting behavior.

H3b: Infrastructure availability positively moderates the relationship between environmental attitude and waste sorting behavior.

**Figure 1:**
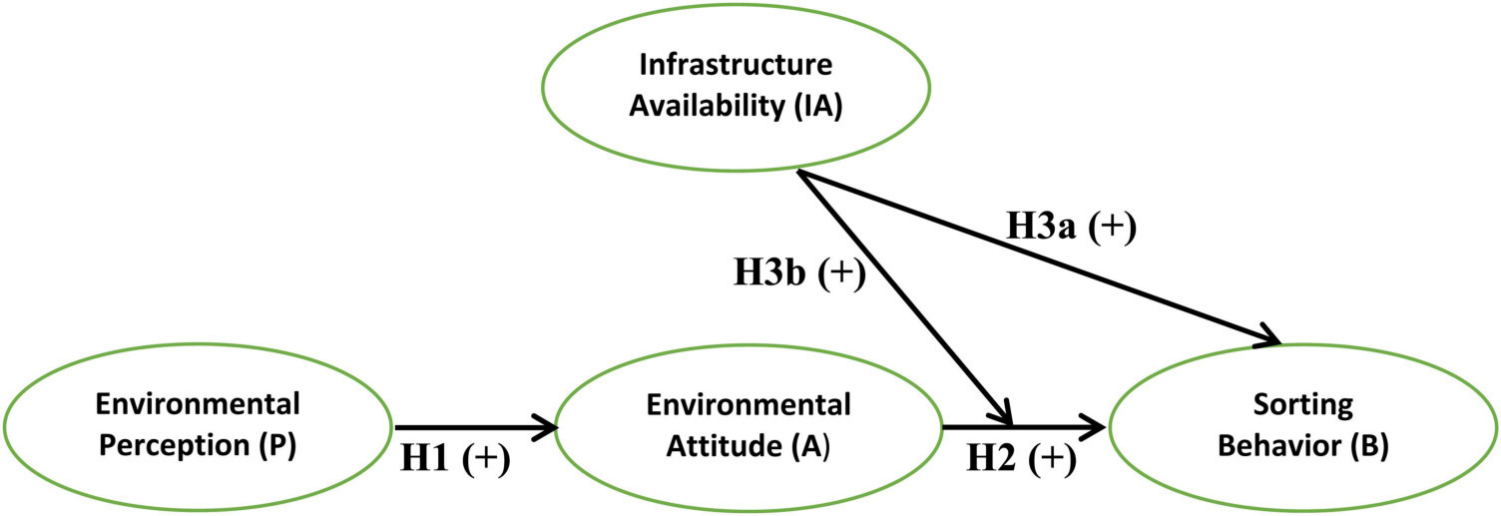
Proposed conceptual framework based on TPB and ABC model.

The conceptual model is presented in Figure 1.

By testing these hypotheses using PLS-SEM on survey data from 460 participants, the study offers three core contributions: (1) theoretically, it elucidates the attitude–behavior gap in an under-researched event context and tests the competing predictions of TPB and the ABC model regarding infrastructure's role; (2) methodologically, it tests simultaneous direct and moderating roles of infrastructure using robust PLS-SEM techniques; (3) practically, it shifts management focus from awareness-raising to optimizing physical environments and reducing friction costs, with specific implications for event organizers in developing countries.

## 2 Research methodology

1. Research Design and Conceptual Model: This study employs a quantitative approach to test a model based on the integration of the Theory of Planned Behavior (TPB) and the Attitude-Behavior-Context (ABC) framework. The proposed model consists of four constructs: Environmental Perception (P), Attitude (A), Infrastructure Availability (IA), and Waste Sorting Behavior (B). The primary hypotheses include:

H1, H2: The positive impacts of P → A and A → B.

H3a, H3b: The direct positive impact of IA → B and the moderating role of IA on the A → B relationship.

2. Measurement and Data Collection: Data were collected using a convenience sampling method at mass sport events in Hai Phong, Bac Giang, and Thanh Hoa. A total of 460 valid responses were included in the final analysis. A 5-point Likert scale was adapted from Stern et al. (11) and Ajzen (10). Following a preliminary assessment based on factor loading coefficients (Outer loadings >0.5), the remaining number of items for each construct is as follows: P (7 items), A (8 items), IA (3 items: IA1, IA2, IA5), and B (3 items: B1, B3, B5).
3. Data Analysis: The study utilizes Partial Least Squares Structural Equation Modeling (PLS-SEM) via SmartPLS 3.0 software, conducted in two stages:

Measurement Model Assessment: Testing for reliability (Cronbach's Alpha, CR > 0.7), convergent validity (AVE > 0.5, Loadings > 0.7), and discriminant validity (Fornell-Larcker criterion, HTMT < 0.85).

Structural Model Assessment: Evaluating coefficients such as *R*^2^, *f*^2^, and *Q*^2^. Hypotheses are tested using the Bootstrapping technique (5,000 resamples). A hypothesis is accepted when *T* > 1.96 (*p* < 0.05).

4. Common Method Bias (CMB) Control: To minimize CMB, the questionnaire was designed with anonymity and structural separation of constructs. Empirical testing indicated that all inner VIF values were below 3.3 (see Table 7), confirming the absence of serious common method bias (29).
5. Sample size justification: Sample size justification: Following PLS-SEM guidelines (30, 31), *n* = 460 is more than adequate. The 10-times rule requires only 30 observations (maximum three paths to Behavior). Inverse square root method confirms power >0.80 for detecting *β* ≥ 0.10 at *α* = 0.05. Thus, the sample size is sufficient.
6. Model specification

Measurement equations (reflective):

P=λ1P1+…+λ7P7+εP

A=λ1A1+…+λ8A8+εA

IA=λ1IA1+λ2IA2+λ3IA5+εIA

B=λ1B1+λ2B3+λ3B5+εB

Structural equations:

A=β1P+ζA

B=β2A+β3IA+β4(A×IA)+ζB

## 3 Results and discussion

### 3.1 Sample characteristics and the status of source waste sorting among participants at mass sport events in Vietnam

#### 3.1.1 Sample characteristics

The study's survey sample consisted of 460 valid participants, collected from mass sport events across three Vietnamese provinces/cities: Hai Phong, Bac Giang, and Thanh Hoa. The descriptive statistics are presented in Table 1.

**Table 1:** Sample characteristics (*N* = 460).

Characteristic	Classification	Frequency (*n*)	Percentage (%)
Gender	Male	288	62.6
	Female	172	37.4
Age group	Under 23 (12–22)	84	18.3
	23–44	185	40.2
	45–60	143	31.1
	Over 60	48	10.4
Highest education	No formal education	1	0.2
	Primary school	8	1.7
	Secondary/High school	281	61.1
	College/Undergraduate	160	34.8
	Postgraduate	10	2.2
Average monthly income	<200 USD	95	20.7
	200–400 USD	223	48.5
	400–600 USD	106	23.0
	600–800 USD	27	5.9
	>800 USD	9	2.0
Interview location	Thanh Hoa	160	34.8
	Hai Phong	150	32.6
	Bac Giang	150	32.6
Respondent type	Residents/Spectators	310	67.4
	Athletes	150	32.6

The study sample consisted of 460 valid participants from Hai Phong, Bac Giang, and Thanh Hoa. Males accounted for 62.6%, higher than females (37.4%). The 23–44 age group represented the largest proportion (40.2%), followed by those aged 45–60 (31.1%), indicating that the sample was concentrated in the working-age population.

In terms of education, most participants had a high school level (61.1%) or college/university degrees (34.8%). Income was mainly at a low to middle level, with nearly half earning between 200 and 400 USD per month (48.5%). Notably, local residents/spectators made up the majority (67.4%), compared to athletes (32.6%), suggesting the sample was skewed toward the audience group.

#### 3.1.2 Status of source household solid waste (HSW) sorting among participants at mass sport events in Vietnam

A face-to-face survey of 460 participants at mass sport events in Hai Phong, Bac Giang, and Thanh Hoa was conducted to assess whether they sorted waste at the point of generation.

The results presented in Table 2 indicate that the rate of source waste sorting reached only 15.87%, while the vast majority of participants (84.13%) did not engage in this behavior. This significant disparity provides clear empirical evidence of the “attitude-behavior gap” in the context of mass sport events. Although participants may possess positive environmental awareness, this has not yet translated into specific actions under field conditions. This finding aligns with the arguments of Kollmuss and Agyeman (32) regarding the inconsistency between environmental awareness and behavior. Furthermore, the potential for social desirability bias in self-reported data must be considered (33) implying that the actual level of behavioral implementation might be even lower than recorded.

**Table 2:** Status of source HSW sorting among participants at mass sport events in Vietnam (*n* = 460).

Content	Sorting performed	%	Sorting not performed	%
Status of HSW sorting at mass sport events	73	15.87	**387**	**84.13**

Bold values indicate the majority response category (“Sorting not performed”).

More importantly, these results underscore the role of contextual factors, particularly Infrastructure Availability. A lack of sorting bins, inconvenient placement, or insufficient guidance can disrupt the transformation of intention into action. This reinforces the perspectives of Ajzen (10) and Steg and Vlek (34) that environmental behavior is simultaneously influenced by both individual factors and external supporting conditions. This situation also reflects the general state of mass sport event organization in Vietnam, where source waste management has not received adequate attention compared to other technical aspects of event management.

### 3.2 Measurement model assessment

#### 3.2.1 Reliability and convergent validity

Prior to testing the structural model and hypotheses, the measurement model was evaluated to ensure the reliability and validity of the scales. Using the PLS-SEM algorithm, several indicators were examined: Outer Loadings to assess indicator reliability; Cronbach's Alpha and Composite Reliability (CR) to evaluate internal consistency; and Average Variance Extracted (AVE) to establish the convergent validity of the latent constructs. The reliability and convergent validity results are presented in Table 3.

**Table 3:** Reliability and convergent validity of the scales (*n* = 460).

Latent constructs	Indicators	Loadings	Cronbach's alpha (*α*)	Composite reliability (CR)	Average variance extracted (AVE)
Environmental perception (P)	P1–P7	0.637–0.886	0.899	0.911	0.596
Environmental attitude (A)	A1–A8	0.661–0.922	0.922	0.935	0.647
Infrastructure availability (IA)	IA1, IA2, IA5	0.671–0.862	0.722	0.842	0.643
Sorting behavior (B)	B1, B3, B5	0.759–0.908	0.826	0.892	0.735

##### 3.2.1.1 Internal consistency reliability

The analysis results show that Cronbach's Alpha values range from 0.722 to 0.922, and Composite Reliability (CR) values range from 0.842 to 0.935. All indicators exceed the recommended threshold of 0.70, indicating high reliability and ensuring the internal consistency of the constructs within the model.

##### 3.2.1.2 Convergent validity

The Average Variance Extracted (AVE) for the latent constructs ranges from 0.596 to 0.735, all of which exceed the minimum threshold of 0.50 suggested by Hair et al. (30). These results confirm that the indicators effectively explain the variance of their respective latent constructs, thereby ensuring the convergent validity of the measurement scales.

##### 3.2.1.3 Indicator refinement

During the measurement model assessment stage, indicators IA3, IA4, B2, and B4 were removed due to outer loadings below 0.50. This refinement was necessary to optimize AVE values and improve the structural model's fit. Notably, indicators IA5 (0.671) and P4 (0.637) were retained despite having loadings below 0.70. This decision was based on the criteria of Hair et al. (30), as these indicators remain theoretically significant, and their retention does not cause the AVE of their respective constructs to fall below the 0.50 threshold.

To provide an overview of the central tendencies and dispersion of the four latent constructs, Table 4 reports the composite means and standard deviations for Environmental Perception, Environmental Attitude, Infrastructure Availability, and Sorting Behavior.

**Table 4:** Descriptive statistics of latent constructs (*N* = 460).

Latent construct	Mean	Standard deviation
Environmental perception (P)	4.06	1.17
Environmental attitude (A)	4.50	0.67
Infrastructure availability (IA)	4.16	0.65
Sorting behavior (B)	2.64	1.35

Table 4 presents the descriptive statistics of the four latent constructs. The mean attitude score was 4.50, while the mean behavior score was only 2.64, confirming the pronounced attitude-behavior gap observed in this study.

#### 3.2.2 Discriminant validity

After establishing convergent validity, discriminant validity was tested to ensure that each latent construct in the model is unique and does not conceptually overlap with others. This study employed two of the most rigorous contemporary approaches: the Fornell-Larcker criterion and the Heterotrait-Monotrait ratio (HTMT). The discriminant validity results based on the Fornell-Larcker criterion are presented in Table 5.

**Table 5:** Discriminant validity results (Fornell-Larcker criterion).

Constructs	A	B	IA	A × IA	P
Environmental attitude (A)	**0.804**				
Sorting behavior (B)	0.116	**0.857**			
Infrastructure availability (IA)	−0.074	0.170	**0.802**		
Interaction (A × IA)	0.043	−0.001	0.002	**1.000**	
Environmental perception (P)	0.399	0.309	0.034	0.033	**0.772**

Values in bold on the diagonal are the square root of the average variance extracted (AVE); other values represent the correlations between constructs.

##### 3.2.2.1 Regarding the Fornell-Larcker criterion

The results in Table 4 show that the square root of the AVE for each construct is greater than all correlation coefficients between that construct and others in the model. This demonstrates that each latent variable explains the variance of its own indicators better than the variance shared with other variables, perfectly satisfying the criteria established by Fornell and Larcker (35).

##### 3.2.2.2 Regarding the HTMT index

**Figure 2:**
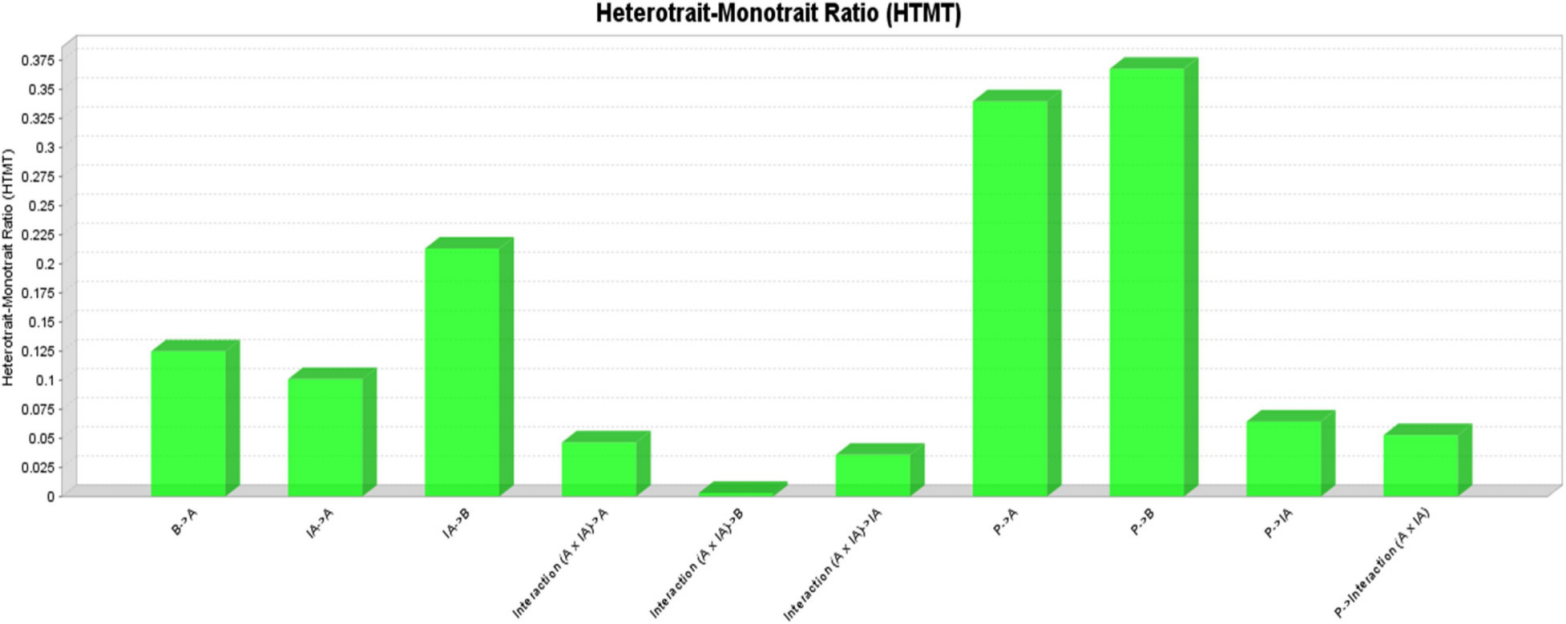
Heterotrait-Monotrait ratio (HTMT) results.

Based on the results in Figure 2, all HTMT ratios between pairs of constructs fall below the rigorous threshold of 0.85, with the highest recorded value being 0.354 (between Environmental Perception and Sorting Behavior). These findings provide solid empirical evidence confirming the distinctiveness of the research constructs, meeting the advanced standards proposed by Hair et al. (30).

### 3.3 Structural model and hypothesis testing

#### 3.3.1 Model fit and explanatory power assessment

To evaluate the quality of the structural model, indicators of explanatory and predictive power were examined. These values were extracted using the PLS-SEM algorithm and the Blindfolding technique in SmartPLS 3.0 software.

##### 3.3.1.1 Coefficient of determination (*R*^2^)

The results indicate that Environmental Perception (P) explains 15.9% of the variance in Environmental Attitude (A). Although the independent variables explain only 4.5% of the variance in Sorting Behavior (B), this result is considered acceptable in empirical studies of human psychology and behavior in real-world settings, where behavior is often governed by complex contextual factors beyond the model (30).

##### 3.3.1.2 Predictive relevance (*Q*^2^)

The Stone-Geisser indicator (*Q*^2^) shows that the values for both Attitude (0.090) and Behavior (0.026) are greater than zero. According to the criteria of Henseler et al. (36), this confirms that the model possesses predictive relevance for the endogenous variables.

##### 3.3.1.3 Effect size (*f*^2^)

Based on Cohen's (1988) criteria, Environmental Perception (P) has a medium effect on Attitude (*f*^2^ = 0.189). Meanwhile, the impacts on Sorting Behavior (B) are characterized as small (A → B: 0.017; IA → B: 0.034), and the moderating effect is negligible (*f*^2^ = 0.000). This further confirms that at mass sport events, the transition from awareness to actual behavior remains a challenging process influenced by numerous structural constraints.

#### 3.3.2 Direct hypotheses and moderating effect testing

**Figure 3:**
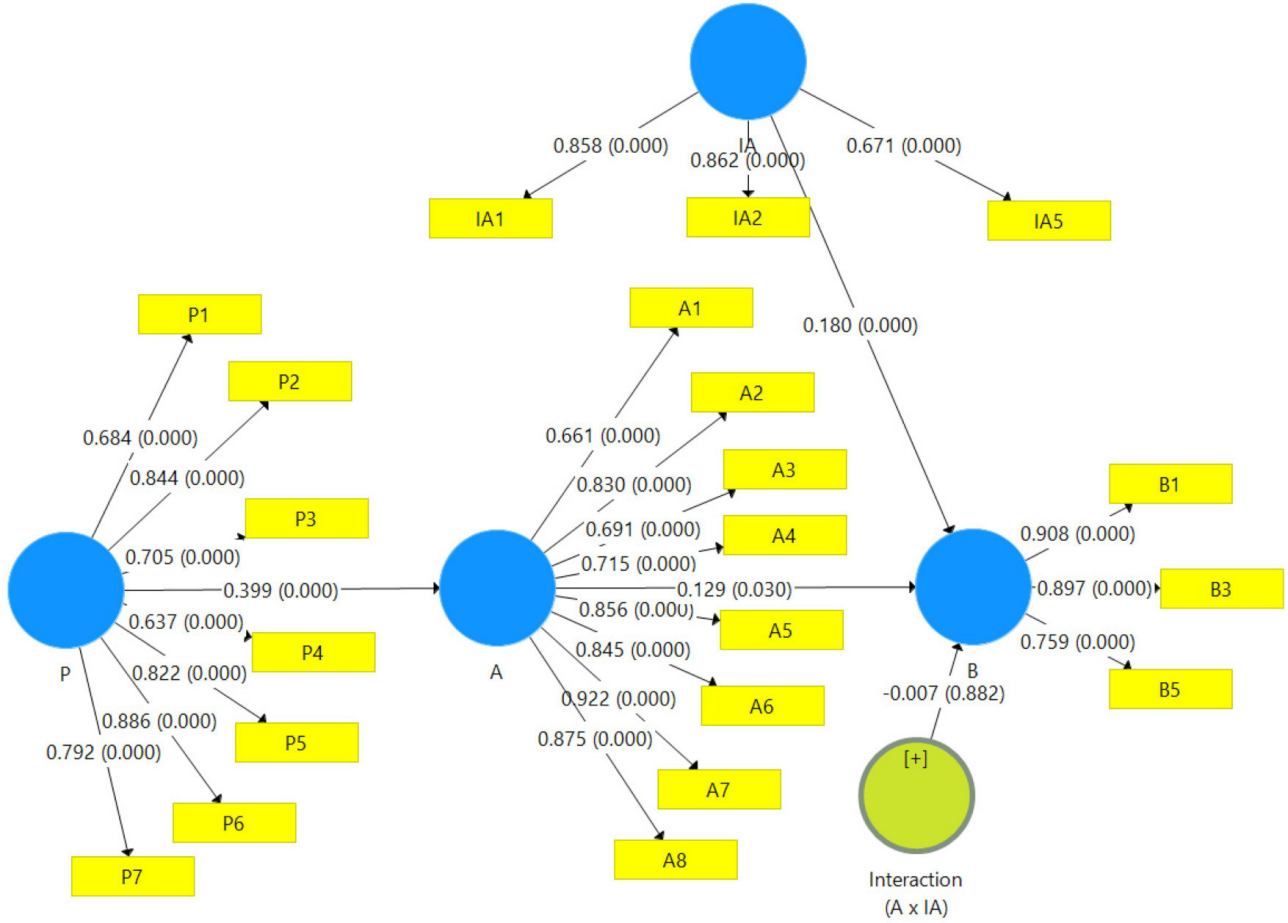
PLS-SEM structural model results showing path coefficients and *p*-values.

To test the proposed hypotheses, a bootstrapping procedure with 5,000 resamples was employed. Table 6 reports the path coefficients (*β*), *T-*statistics, *p*-values, bias-corrected and accelerated (BCa) 95% confidence intervals, and inner VIF values for all predictors. Detailed results are presented in Figure 3 and Table 7.

**Table 6:** Results of *R*^2^, *f*^2^ và *Q*^2^, and *Q*^2^ assessment.

Endogenous	*R* ^2^	*R*^2^ Adjusted	*Q* ^2^	Predictors	*f* ^2^
Environmental attitude (A)	0.159	0.157	0.090	Environmental perception (P)	0.189
Sorting behavior (B)	0.045	0.039	0.026	Environmental attitude (A)	0.017
				Infrastructure availability (IA)	0.034
				Interaction (A × IA)	0.000

**Table 7:** Hypothesis testing results for the structural model.

Hyp.	Relationship	Coeff. (*β*)	*T*-stats	*P*-values	95% CI (BCa)	VIF	Decision
H1	Environmental perception (P) → attitude (A)	0.399	12.111	0.000	[0.336; 0.459]	1.000	Supported
H2	Environmental attitude (A) → behavior (B)	0.129	2.454	0.014	[0.007; 0.218]	1.007	Supported
H3a	Infrastructure availability (IA) → behavior (B)	0.180	4.367	0.000	[0.092; 0.249]	1.006	Supported
H3b	A × IA → behavior (B)	−0.007	0.147	0.883	[−0.104; 0.079]	1.002	Rejected

All inner VIF values are below 3.3, indicating no serious multicollinearity and confirming the absence of common method bias (29).

The structural model assessment, as illustrated in Figure 3, provides a comprehensive visualization of the hypothesized relationships. The values on the internal paths represent the standardized path coefficients (*β*), with their corresponding *p*-values indicated in parentheses. For the outer model, the values reflect the outer loadings and their respective *p*-values, all of which demonstrate high statistical significance (*p* < 0.001). This final model confirms that Infrastructure Availability (IA), Environmental Perception (P), and Attitude (A) are significant predictors of Sorting Behavior (B), while the interaction effect (A × IA) is empirically rejected.

##### 3.3.2.1 Direct effects

Hypothesis H1, which posited a positive effect of Environmental Perception on Attitude, was supported (*β* = 0.399, *T* = 12.111, *p* < 0.001, 95% CI [0.336; 0.459]). This represents the strongest relationship in the model, confirming that perception is a key antecedent of attitude.

Hypothesis H2 predicted a positive effect of Attitude on Behavior. This relationship was also supported, albeit with a much smaller effect size (*β* = 0.129, *T* = 2.454, *p* = 0.014, 95% CI [0.007; 0.218]), providing empirical evidence of the attitude-behavior gap.

Hypothesis H3a, which proposed a direct positive effect of Infrastructure Availability on Behavior, was supported (*β* = 0.180, *T* = 4.367, *p* < 0.001, 95% CI [0.092; 0.249]), and its magnitude was larger than that of H2, indicating that physical infrastructure is a stronger direct predictor of sorting behavior than individual attitude.

##### 3.3.2.2 Moderating effect

Hypothesis H3b predicted that Infrastructure Availability positively moderates the relationship between Attitude and Behavior. The interaction term (A × IA) was not statistically significant (*β* = –0.007, *T* = 0.147, *p* = 0.883, 95% CI [–0.104; 0.079]). Thus, H3b is rejected.

This finding suggests that in the context of mass sport events in Vietnam, infrastructure does not act as a catalyst that strengthens the translation of attitude into behavior. Instead, it operates as a direct structural determinant.

##### 3.3.2.3 Collinearity assessment

As shown in Table 6, all inner VIF values were below 3.3 (ranging from 1.000 to 1.007), confirming that multicollinearity is not a concern and that common method bias is unlikely (29).

## 4 Discussion

The research findings provide significant empirical evidence regarding the transition mechanism from perception to solid waste sorting behavior at the source within the context of mass sport events in Vietnam. These discoveries not only reinforce existing theories but also extend the understanding of contextual factors' roles in the practical conditions of a developing nation.

### 4.1 The disconnect between perception and actual behavior

The study confirms a strong positive relationship from environmental perception to attitude (H1: *β* = 0.399; *p* < 0.001). This aligns with classic studies by Kollmuss and Agyeman (32) and Stern (37), affirming that environmental knowledge is the core “raw material” for forming positive attitudes.

However, a paradox emerges as the impact of Attitude on Behavior (H2: *β* = 0.129; *p* < 0.05) is the lowest in the model. This clearly reflects the “Attitude-Behavior Gap.” Although participants express rational support for environmental protection, the translation of “willpower” into “action” suffers a significant fracture. This finding validates (10) argument in the Theory of Planned Behavior (TPB): attitude is merely a necessary condition; actual behavior is heavily dictated and controlled by contextual factors and situational constraints.

### 4.2 Infrastructure: from supporting factor to direct determinant

One of the most critical contributions of this study is the confirmation that Infrastructure Availability (IA) has a direct and potent impact on behavior (H3a: *β* = 0.180; *p* < 0.001), surpassing the influence of individual attitude.

This result supports the perspective of Steg and Vlek (34) that in public settings, situational factors (such as the availability of sorting bins and visual signage) play a more decisive role than individual will. At mass sport events, where convenience is prioritized and the window for decision-making is brief, unsynchronized infrastructure renders even the most environmentally conscious individuals unable to sort waste correctly. This explains why the actual sorting rate in the survey was a modest 15.87%, despite a generally positive attitude across the sample.

### 4.3 Theoretical implications of the absent moderating effect

A novel and distinctive finding of this study lies in the rejection of the moderating role of infrastructure on the Attitude-Behavior relationship (H3b: *p* = 0.883). Unlike studies in developed countries, such as those by Guagnano (23) or Verplanken and Aarts (38), which view infrastructure as a “catalyst” that modifies the intensity of attitude's impact, the results in the Vietnamese context suggest that infrastructure functions as a “deterministic” independent variable.

This carries significant theoretical weight: in the context of rudimentary waste management infrastructure at mass sport events, Infrastructure Availability is not merely an “adjuster” but a “physical threshold.” In the absence of sorting bins, an individual's positive attitude is substantially weakened. This represents an important extension of TPB when applied to developing contexts: Contextual factors can act as direct structural barriers rather than just supporting or moderating roles.

## 5 Implications and recommendations

### 5.1 Theoretical implications

This study contributes to extending the application of the Theory of Planned Behavior (TPB) within the context of environmental management at mass sport events.

#### 5.1.1 First

The research provides empirical evidence of the persistent “attitude-behavior gap.” The limited impact of attitude on actual behavior suggests that traditional behavioral models, which primarily focus on internal psychological factors, must integrate contextual elements to enhance predictive power in real-world scenarios.

#### 5.1.2 Second

The finding regarding the direct, rather than moderating, role of Infrastructure Availability (IA) represents a novel contribution. It indicates that in highly organized event environments with significant time pressure, external factors are not merely “catalysts” but can function as independent “determinant factors.” This opens a new research direction for reclassifying the roles of contextual factors in social psychology models.

### 5.2 Practical implications and recommendations

Based on the empirical findings, the study proposes several strategic solutions to bridge the gap between perception and behavior:

Shifting focus from “Cognitive Communication” to “Behavioral Design”: While educational measures remain foundational, event organizers should avoid the over-expectation that awareness changes will automatically result in behavioral changes. The focus should shift toward designing event environments where the correct behavior (waste sorting) becomes the easiest and the “default” choice.

Prioritizing “Visual Infrastructure” Investment: Given that infrastructure has the strongest direct impact, mass sport events must ensure that sorting bin systems are arranged according to three principles: (i) Availability (high density); (ii) Convenience (placed along primary traffic flows); and (iii) Intuition (using color codes and illustrations instead of text-heavy labels).

Utilizing “Nudge” Strategies in Event Management: Behavioral prompting techniques should be leveraged on-site, such as using floor markings that lead to bin locations or deploying volunteers to assist with sorting at “waste hotspots.” These “nudges” help participants make quick and accurate decisions in crowded, high-pressure environments.

Integrating Environmental Management into Organizational Protocols: State management agencies for mass sports should incorporate “waste management at the source” as a mandatory criterion in the licensing process and as a key performance indicator (KPI) for evaluating the success of mass sport events.

## 6 Conclusion

This study elucidates the mechanism underlying solid waste sorting behavior at the source among participants in mass sport events in Vietnam. Through Partial Least Squares Structural Equation Modeling (PLS-SEM) analysis, three primary conclusions have been drawn:

First: The existence of a significant “attitude-behavior gap.” While environmental perception serves as a powerful antecedent for forming positive attitudes (*β* = 0.399), these attitudes struggle to translate into actual action (*β* = 0.129). The low rate of self-reported sorting behavior (15.87%) further demonstrates a distinct fracture between individual willpower and physical action in an event setting.

Second: Infrastructure plays a “deterministic” role in behavior. With a direct impact magnitude (*β* = 0.180) surpassing that of individual attitude, infrastructure is not merely a supporting condition but a physical threshold that determines behavioral execution. When infrastructure is inconvenient, internal psychological factors are substantially undermined.

Third: The moderating effect of infrastructure is statistically non-significant. This indicates that in developing nations with rudimentary waste management systems, infrastructure functions as an independent variable with a direct impact, rather than serving as a catalyst as suggested in models developed in more advanced economies.

Contributions and Future Directions: The study emphasizes the urgent need to shift management focus from purely cognitive education to behavioral environment design and investment in visual infrastructure. This shift is key to narrowing the behavioral gap and achieving the goal of sustainable sport event organization in Vietnam. Future research could expand upon this model by examining the roles of social pressure or mandatory administrative regulations to further refine behavioral predictability.

## 7 Limitations and future research

Despite the significant findings, this study possesses certain limitations that offer avenues for future research:

Geographical Scope: The current research data focuses on three specific provinces in the Northern and Central regions of Vietnam. Given the cultural nuances and variations in waste management infrastructure across regions, future studies should expand the survey scale to major metropolitan areas to enhance the generalizability of the findings.

Explanatory Power of the Model: The coefficient of determination for the behavior variable remains low (*R*^2^ = 0.045), indicating that a substantial portion of the variance in waste sorting behavior at events remains unexplained by the current variables. Subsequent research should integrate additional constructs from the TPB framework, such as Subjective Norms, or extended factors like Habits, Opportunity Costs, and Incentives to bolster the model's explanatory power.

Measurement Methodology: This study relies primarily on self-reported data, which may be susceptible to social desirability bias. The remarkably low rate of actual reported behavior (15.87%) suggests that future research should employ mixed methods or field observations. Delving deeper through qualitative approaches will help elucidate the complex psychological and situational barriers that quantitative methods may not fully capture.

Additionally, the attitude-behavior gap reported in this study should be interpreted with caution. Attitude items used “importance/agreement” anchors (e.g., “strongly disagree—strongly agree”), whereas behavior items used frequency anchors (e.g., “never—always”). This difference in scale wording may partly contribute to the observed mean discrepancy. Future research should employ consistent scale anchors or, ideally, combine self-reported behavior with objective measures such as direct observation or smart bin tracking.

## Data Availability

The raw data supporting the conclusions of this article will be made available by the authors without undue reservation.
